# Ischemia-Modified Albumin (IMA) Is Associated with Poor Survival in Patients with Newly Diagnosed Idiopathic Pulmonary Fibrosis (IPF): A Pilot Study

**DOI:** 10.3390/antiox13030278

**Published:** 2024-02-25

**Authors:** Angelo Zinellu, Stefano Zoroddu, Simona Fois, Sabrina Mellino, Chiara Scala, Erika Virdis, Elisabetta Zinellu, Salvatore Sotgia, Panagiotis Paliogiannis, Arduino A. Mangoni, Ciriaco Carru, Pietro Pirina, Alessandro G. Fois

**Affiliations:** 1 Department of Biomedical Sciences, University of Sassari, 07100 Sassari, Italy; szoroddu@uniss.it (S.Z.); s.mellino@studenti.uniss.it (S.M.); ssotgia@uniss.it (S.S.); carru@uniss.it (C.C.); 2 Department of Respiratory Diseases, University Hospital Sassari (AOU), 07100 Sassari, Italy; s.fois57@studenti.uniss.it (S.F.); chiara.scala.cs93@gmail.com (C.S.); virdiserika@gmail.com (E.V.); elisabetta.zinellu@aouss.it (E.Z.); pirina@uniss.it (P.P.); 3 Department of Medicine, Surgery and Pharmacy, University of Sassari, 07100 Sassari, Italy; ppaliogiannis@uniss.it; 4 Discipline of Clinical Pharmacology, College of Medicine and Public Health, Flinders University, Bedford Park, SA 5042, Australia; arduino.mangoni@flinders.edu.au; 5 Department of Clinical Pharmacology, Flinders Medical Centre, Southern Adelaide Local Health Network, Bedford Park, SA 5042, Australia

**Keywords:** ACB test, IMA, IPF, survival

## Abstract

There are increasing efforts to better predict adverse outcomes for idiopathic pulmonary fibrosis (IPF). Our aim was to assess the prognostic potential of ischemia-modified albumin (IMA), an established circulating marker of ischemia and, more recently, oxidative stress, in a cohort of 56 IPF patients recruited between 2015 and 2023 at the University of Sassari, Italy. Demographic and functional parameters and serum IMA concentrations were measured at baseline. Non-survivors had significantly higher IMA concentrations vs. survivors (508 ± 64 vs. 474 ± 42 mABSU, respectively; *p* = 0.035). The Kaplan–Meier analysis showed a significant association between higher IMA values and poor survival (HR: 3.32, 95% CI from 1.06 to 10.4, *p* = 0.039). In the Cox regression analysis, this association remained significant after adjusting for the force expiratory volume at 1 s, the total lung capacity, lymphocyte count, and pharmacological treatment (HR: 1.0154, 95% CI from 1.0035 to 1.0275, *p* = 0.01). IMA, an oxidative stress biomarker measurable using relatively simple and available methods, is independently associated with mortality in IPF. Therefore, its determination may enhance risk stratification and treatment decisions. Prospective studies involving larger cohorts are needed to confirm this association and to endorse the use of IMA in routine practice.

## 1. Introduction

Idiopathic pulmonary fibrosis (IPF) is a progressive interstitial lung disease characterized by fibrotic changes leading to impaired gas exchange and ultimately respiratory failure [1,2]. While extensive research has shed light on its pathogenesis, IPF remains a challenging and often fatal condition with limited therapeutic options [3]. Among the various contributing factors to IPF progression, oxidative stress has emerged as a critical player [4,5]. Oxidative stress, characterized by a dysregulation in the equilibrium between the generation of reactive oxygen species (ROS) and antioxidative defense mechanisms, plays a pivotal role in the onset and advancement of IPF. The excessive production of ROS contributes to cellular damage, inflammation, and fibrosis in the lung tissue of IPF patients. The lung, being exposed to higher oxygen levels compared to other tissues, exhibits heightened sensitivity to oxidative stress [6]. External factors, such as exogenous oxidants and pollutants, amplify the production of oxidants and trigger inflammatory cells, prompting the generation of free radicals [7]. Multiple enzymes are involved in the production of ROS, including NADPH oxidase (NOXs), xanthine oxidase, nitric oxide synthase (NOS), and the mitochondrial electron transport chain [7,8]. Additionally, ROS are generated during the metabolic breakdown of various drugs and xenobiotics [7]. ROS, encompassing molecules such as superoxide (O_2_^−)^ and hydrogen peroxide (H_2_O_2_), serve as essential components in the body defense system by eliminating microbes in phagocytic cells. However, while lower levels of ROS have several important physiological roles as signaling molecules, excessive ROS levels can be toxic and may trigger pathological conditions, including fibrosis [7,8]. Within the spectrum of oxidative stress biomarkers, ischemia-modified albumin (IMA) has emerged as a promising biomarker of oxidative stress [9,10]. IMA reflects the alteration of serum albumin due to the influence of reactive oxygen species that induces structural changes in the N-terminal region of proteins, which may be measured by spectrophotometry using the albumin cobalt binding (ACB) test [11]. It is an altered form of human serum albumin (HSA) characterized by a diminished affinity for metals compared to conventional HSA. HSA contains four recognized metal-binding sites: the N-terminus site (NTS), sites A and B, and the cysteine-34 residue (Cys-34). The process of IMA formation is attributed to two proposed mechanisms: ROS-induced NTS degradation and a conformational change induced by free fatty acids (FFAs) [12,13]. Under the ROS-induced NTS degradation theory, oxidative stress triggers the separation of a dipeptide from the NTS during ischemic conditions. This leads to a truncated form of HSA, causing reduced affinity for metal binding. However, studies on kinetics and sequencing have suggested that IMA formation is transient in nature rather than representing a structural alteration [12,13,14,15]. On the other hand, according to the FFA-induced conformational change model, the interaction between FFAs and HSA prompts conformational alterations in the HSA structure, subsequently decreasing the affinity of sites A and B for metal binding. This model finds support in biophysical studies and the observation of elevated circulating levels of FFAs in conditions associated with oxidative stress and ischemia [16,17,18]. The initial discovery of elevated circulating IMA levels during cardiac ischemia was reported by Bar-Or et al. [11], who introduced the ACB test for IMA measurement. Subsequent research expanded this finding, revealing that serum IMA concentrations increased in various pathological conditions linked to oxidative stress. As a result, it has been regarded not only as a biomarker of ischemia but also as an indicator of oxidative stress within diverse pathological contexts [19,20,21,22,23]. This study aims to explore the possible association between the serum concentrations of IMA and adverse clinical outcomes in patients diagnosed with IPF and, therefore, provide initial mechanistic insights into the factors mediating the link between oxidative stress and disease progression in this group.

## 2. Materials and Methods

### 2.1. Study Population

We recruited a consecutive series of 56 patients with newly diagnosed IPF attending the Respiratory Unit of the University of Sassari from 2016 to 2023. The study was approved by the ethics committee of the University Hospital of Cagliari (Approval No. 2262/CE-17/11/2015). A written consent was provided by all participants; the study was conducted in accordance with the Declaration of Helsinki. IPF was diagnosed according to established guidelines [1]. The evaluation of high-resolution computed tomography (HRCT) scans and lung biopsy specimens was conducted by two experienced radiologists and two experienced pathologists, respectively. Each case was discussed in a multidisciplinary meeting attended by local experts in respiratory care, pathology, and radiology with a focus on interstitial lung diseases.

Forced expiratory volume in 1 s (FEV1), forced vital capacity (FVC), total lung capacity (TLC), and diffusing capacity of the lungs for carbon monoxide (DLCO) were measured in each patient. The results are expressed as percentages of the predicted values (FEV_1_%, FVC%, and DLCO%, respectively), according to the existing guidelines [24]. The exclusion criteria included acute IPF exacerbations, comorbidities such as cancer, bleeding, and severe liver or kidney impairment.

### 2.2. Ischemia-Modified Albumin

Blood was collected after an overnight fast and centrifuged at 4 °C and 1500× *g* for 10 min to separate the serum, which was stored at −80 °C until analysis.

The ACB test was used to measure IMA [11]. Fifty microliters of 0.1% cobalt chloride (CoCl_2_, 6H_2_O) were added to the serum, which, after mixing, was incubated for 10 min. Afterward, 50 μL of 1.5 mg/mL dithiothreitol was added and incubated for 2 min after the mixing procedure. Finally, 1 mL of a 0.9% NaCl solution was added to stop the reaction. A blank solution was prepared with the same method but using distilled water instead of dithiothreitol. The absorbance was assessed spectrophotometrically at 470 nm, and results are expressed as milli-absorbance units (mABSU).

### 2.3. Statistical Analysis

The variable distribution was assessed using the Kolmogorov–Smirnov test and the data are presented as the mean values (mean ± SD) or median values (median and IQR). The between-group differences in the continuous variables were analyzed using an unpaired Student’s *t*-test for the parametrically distributed variables or the Mann–Whitney rank-sum test for the non-parametrically distributed variables. The Levene test was used to check the homoscedasticity of the variables. For survival analysis, time zero was defined as the time of the diagnosis of IPF. The survival probability for all parameters was estimated using the means of the Kaplan–Meier curves with the end point being death. For the continuous variables, the median values were used as the cut-off points. Cox proportional-hazards regression was also performed for both univariate and multivariate analyses. The independent association between IMA and survival was assessed after correcting for confounders exhibiting a *p*-value of <0.1 in the univariate analysis (TLC%, FVC%, therapy, and lymphocyte count) by multivariate Cox regression models with backward elimination. MedCalc for Windows, version 19.4.1 64 bit, was used for the statistical analyses (MedCalc Software, Ostend, Belgium).

## 3. Results

A total of 56 IPF patients (44 men and 12 women) were included (Table 1). Twelve patients (21%) died during the study, while forty-four (79%) survived. The mean survival was 34 ± 22 months for the entire patient cohort and was statistically different between survivors and non-survivors (37 ± 22 vs. 21 ± 17 months, respectively; *p* = 0.03). The mean age at diagnosis was 70.3 ± 6.9 years. The mean ages of survivors and non-survivors, 70.7 and 68.8 years, respectively, were similar (*p* = 0.40). There were no significant between-group differences in the body mass index (BMI). Moreover, no significant differences in pre-existing diseases (diabetes, arterial hypertension, cerebrovascular disease, and atrial arrhythmias) were observed between survivors and non-survivors, even if a trend toward an increased frequency of individuals with hypertension was observed in non-survivors (67% vs. 36%, respectively; *p* = 0.06). In the survivor group, 45% of patients were in stage I, 39% in stage II, and 16% in stage III, whereas, in the non-survivor group, 25% of patients were in stage I, 42% in stage II, and 33% in stage III, with a non-significant difference between the two groups (*p* = 0.30). A total of 3 patients did not receive antifibrotic medications, while 24 were treated with nintedanib and 29 with pirfenidone. There were no significant between-group differences in drug utilization (*p* = 0.09). The TLC% was significantly lower in non-survivors than in survivors (66.6 ± 17.6 vs. 78.2 ± 16.2, respectively; *p* = 0.035), and a non-significant trend toward reduced FEV1% and FVC% values was observed in non-survivors (73.0 ± 21.2 vs. 85.7 ± 20.5, *p* = 0.07, and 66.0 ± 17.3 vs. 78.3 ± 19.8, *p* = 0.06, respectively). No significant between-group differences in DLCO (*p* = 0.30) and 6MWT (*p* = 0.25) were observed. Non-survivors had lower lymphocyte values (1.53 ± 0.65 vs. 2.42 ± 0.88 × 10^9^ L, *p*  =  0.002). There were no significant between-group differences in the whole blood count and monocyte and neutrophil counts.

**Figure 1:**
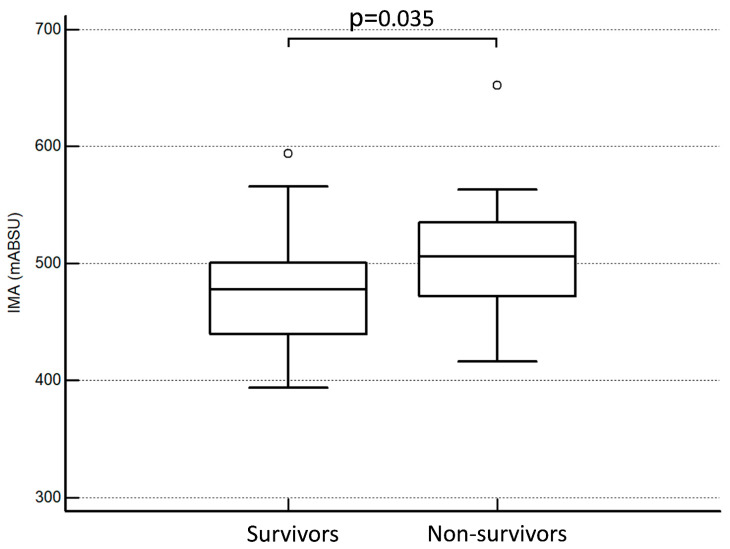
Serum concentration of IMA in survivors and non-survivors.

**Figure 2:**
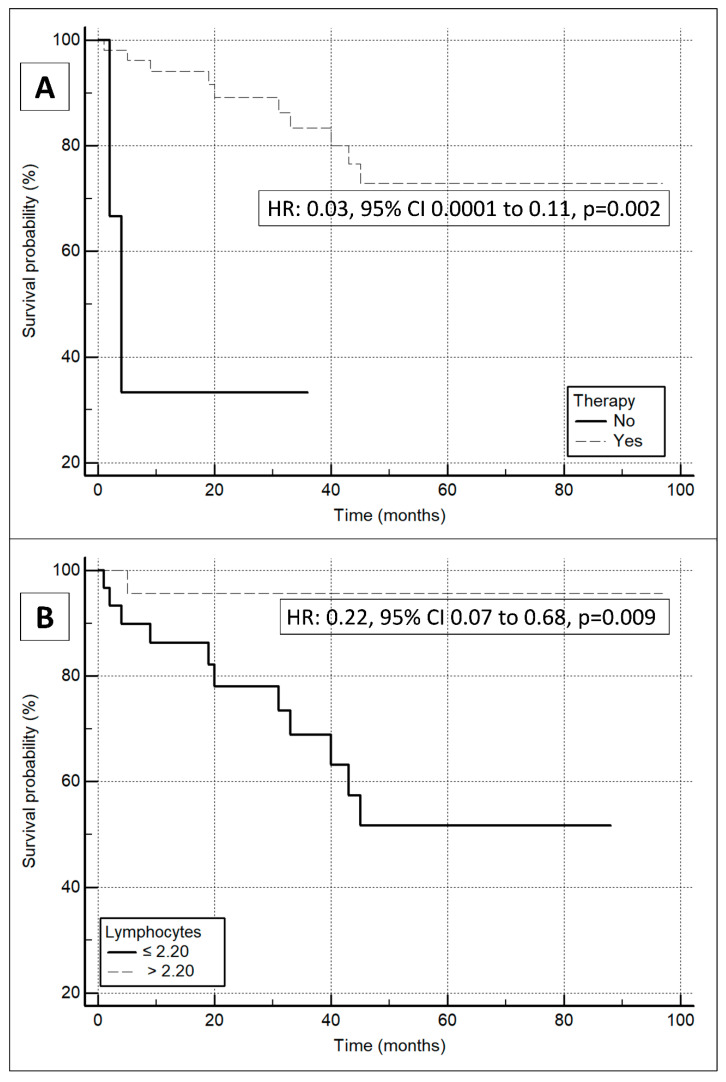
Kaplan–Meier curves for the survival probability of IPF patients according to therapy (**A**) and lymphocyte level (**B**).

**Figure 3:**
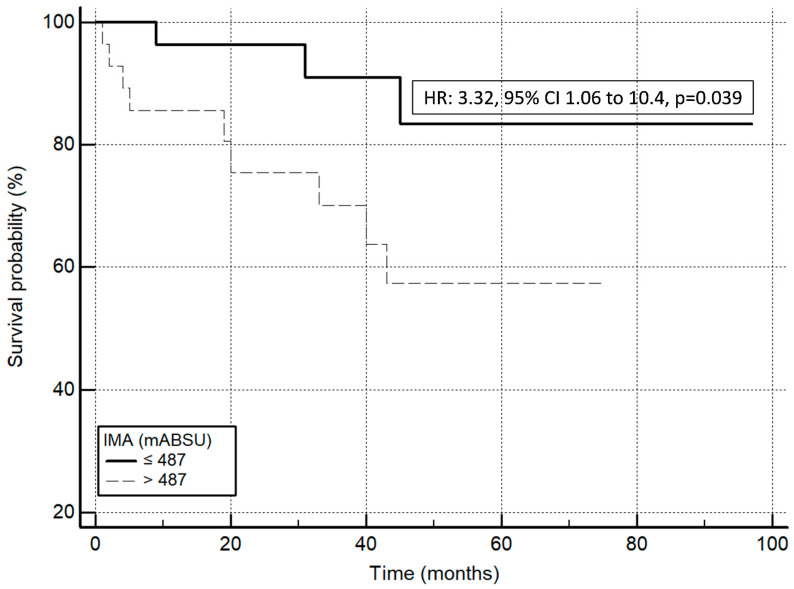
Kaplan–Meier curves for the survival probability of IPF patients according to serum IMA values.

According to Figure 1, the non-survivor group exhibited significantly higher IMA concentrations compared to the survivor group (508 ± 64 vs. 474 ± 42 mABSU, respectively; *p* = 0.035). A Kaplan–Meier analysis was performed for all studied parameters, and significant associations were observed between survival and treatment (HR: 0.03, 95% CI from 0.0001 to 0.11, *p* = 0.002; Figure 2A), and survival and lymphocyte count (HR: 0.22, 95% CI from 0.07 to 0.68, *p* = 0.009; Figure 2B). In addition, there was a significant association between survival and IMA concentrations (HR: 3.32, 95% CI from 1.06 to 10.4, *p* = 0.039, for the group with IMA values ≤ 487; Figure 3).

The univariate Cox regression analysis reported in Table 2 confirmed the associations between survival and lymphocyte count (HR: 0.2671, 95% CI from 0.1110 to 0.6427, *p* = 0.003) and IMA (HR: 1.0154, 95% CI from 1.0035 to 1.0275, *p* = 0.01). Moreover, there was a non-significant association trend between survival and TLC% (HR: 0.9587, 95% CI from 0.9189 to 1.0001, *p* = 0.051), therapy (HR: 0.3993, 95% CI from 0.1432 to 1.1135, *p* = 0.08), and FVC% (HR: 0.9720, 95% CI from 0.9419 to 1.0031, *p* = 0.08). Considering the extensive recruitment period spanning from 2016 to 2023 and the potential impact of storing IMA at −80 °C on its concentration, especially given the limited information available in the literature regarding the effect of storage on IMA values, we conducted a thorough evaluation of this parameter through a univariate Cox regression. Our analysis revealed that the year of sample withdrawal was not significantly associated with survival (HR = 0.85, 95% CI from 0.61 to 1.19, *p* = 0.34). In the multivariate Cox regression analysis with backward elimination (Table 3), the HR for IMA remained significant after adjusting for FVC%, TLC%, lymphocyte count, and therapy (HR: 1.0118, 95% CI from 1.0008 to 1.0230, *p* = 0.036). Inserting the year of sample withdrawal into the multivariate model did not alter the results, indicating that sample storage does not exert a significant influence.

## 4. Discussion

Several studies have suggested that the measurement of structurally modified albumin could assist with monitoring oxidative stress in several clinical conditions, such as renal failure, ischemic heart disease, acute appendicitis, cerebral infarction, and pre-eclampsia [19,20,21,22,23]. It is well known that IPF patients exhibit heightened systemic and tissue oxidative stress levels, influenced by various contributing factors that exacerbate this oxidative burden within the disease context [4,5]. Primarily, the continuous exposure of the lungs to environmental insults, such as cigarette smoke, air pollutants, occupational hazards, and other external sources of oxidants, significantly amplifies oxidative stress levels [4,6]. Additionally, the diseased lung microenvironment in IPF, marked by chronic inflammation, impaired antioxidant defenses, and ongoing tissue injury and repair processes, fosters an environment prone to increased oxidative burden. Dysregulated cellular processes, including dysfunctional mitochondria and altered redox signaling pathways, also contribute to elevated ROS production within lung cells [6].

In our study, we found that IMA was significantly associated with mortality in IPF patients in the Kaplan–Meier survival analysis and Cox regression analysis after correction for FEV1%, TLC%, lymphocyte count, and therapy. In particular, an IMA increase of one unit in mABSU corresponds to a 1.18% elevation in the mortality risk. This observation assumes significance given the range of IMA values in our patient population, which spans from 400 to 650 mABSU. Notably, individuals with higher IMA values exhibit about a 3-fold increased risk of mortality compared to patients with lower values. This association may be explained by several factors. Firstly, increasing the circulating concentrations of IMA reflects an increased degree of systemic oxidative stress. In IPF, where oxidative stress plays a crucial role in disease progression, higher levels of IMA suggest a greater oxidative stress burden, exacerbating tissue damage and fibrosis within the lungs [4,6]. Secondly, the association between elevated IMA and reduced survival could also reflect the presence of a more severe and advanced stage of the disease. IPF is a progressive condition characterized by the excessive deposition of scar tissue in the lungs, and increased IMA concentrations may correlate with the extent of lung injury and diminished lung function, ultimately impacting patient survival. Therefore, the elevated concentrations of IMA in IPF patients likely reflect both the heightened oxidative stress implicated in disease pathogenesis and the disease advanced stage, contributing to reduced patient survival rates.

In addition, our data also indicated a significant association between a reduced lymphocyte count and mortality in IPF. This finding agrees well with recent observations in which IPF subjects showed reduced lymphocyte counts when compared to the healthy controls [25]. In addition, a significant association between a low lymphocyte count and increased risk of both symptom deterioration (evaluated as FVC decline) and mortality has been recently reported [26]. Finally, several combined indexes that include the lymphocyte count, such as the neutrophil-to-lymphocyte ratio (NLR), lymphocyte-to-monocyte ratio (LMR), platelet-to-lymphocyte ratio (PLR), systemic inflammatory response index (SIRI), and aggregate index of systemic inflammation (AISI), have been reported to be associated with a poor survival in IPF [26,27,28,29]. The alterations in these combined indices in IPF patients were correlated with a characteristic inflammatory state in this condition. Patients with IPF exhibited a notable correlation between oxidative stress and inflammation, wherein increased oxidative stress levels coincide with heightened inflammatory responses within the affected lung tissue. Released ROS molecules act as signaling mediators, stimulating the release of pro-inflammatory cytokines and chemokines. This, in turn, attracts immune cells to the site of injury, perpetuating chronic inflammation within the lungs of IPF patients [30,31,32,33]. Moreover, inflammation further fuels oxidative stress by activating additional pathways that generate ROS, creating a feedback loop where oxidative stress and inflammation continuously reinforce each other, contributing to disease progression and lung tissue fibrosis in IPF [30,31,32,33]. Our study is subject to certain limitations, specifically the relatively small sample size and the small number of women (n = 12). While our findings provide valuable insights, the limited number of female participants may impact the generalizability of the results to a broader population. Additionally, in consideration of the single center design, the study predominantly involves a specific ethnic group. Considering the potential influence of ethnicity on health outcomes, caution should be exercised when extrapolating our observations to different ethnicities. Future research endeavors with larger and more diverse cohorts encompassing various ethnic backgrounds are essential to confirm the observed associations and conclusions.

To summarize, our study indicates that IMA, an accessible and cost-effective biomarker, offers valuable prognostic insights and the potential identification of individuals at increased risk of early mortality. However, further studies with larger sample sizes are necessary to confirm the prognostic utility of IMA in predicting mortality among IPF patients and justify its routine use in clinical practice.

## 5. Conclusions

Our study underscores the significance of elevated IMA concentrations as a prognostic marker in IPF. Higher IMA concentrations were consistently associated with poor survival, likely reflecting the increased oxidative stress burden and disease severity in IPF patients. Additionally, the correlation between reduced lymphocyte count and unfavorable prognosis aligns with emerging evidence highlighting the role of inflammation in IPF progression. These findings emphasize the intertwined nature of oxidative stress, inflammation, and disease advancement in IPF pathogenesis, warranting further validation in larger, multicenter cohorts to guide clinical management strategies effectively.

## Figures and Tables

**Table 1:** Demographic, clinical, and laboratory characteristics of the study population.

	Global Cohort (*n* = 56)	Survivors (*n* = 44)	Non-Survivors (*n* = 12)	*p*-Value
Age, years	70.3 ± 6.9	70.7 ± 6.9	68.8 ± 6.8	0.40
Gender (M/F)	45/11	34/10	11/1	0.27
BMI (Kg/m^2^)	27.5 ± 4.0	27.2 ± 3.6	28.4 ± 5.2	0.37
Smoking status, *n* (no/former/yes)	13/42/1	11/32/1	2/10/0	0.71
Diabetes, *n* (no/yes)	46/10	37/7	9/3	0.47
Arterial hypertension, *n* (no/yes)	32/24	28/16	4/8	0.06
Cerebrovascular diseases, *n* (no/yes)	52/4	41/3	11/1	0.86
Atrial arrhythmias, *n* (no/yes)	49/7	40/4	9/3	0.19
GERD, *n* (no/yes)	39/17	30/14	9/3	0.65
FEV_1_ (%)	83.0 ± 21.2	85.7 ± 20.5	73.0 ± 21.2	0.07
FVC (%)	75.7 ± 19.8	78.3 ± 19.8	66.0 ± 17.3	0.06
FEV_1_/FVC (%)	89.3 ± 6.4	89.2 ± 6.8	91.0 ± 4.1	0.39
TLC (%)	75.6 ± 17.0	78.2 ± 16.2	66.6 ± 17.6	0.035
DLCO (%)	53.2 ± 20.6	54.7 ± 22.0	47.4 ± 14.2	0.30
6MWT (meters)	323 ± 173	336 ± 173	266 ± 171	0.25
Stage (I/II/III)	23/22/11	20/17/7	3/5/4	0.30
Therapy (no/nintedanib/pirfenidone)	3/24/29	1/18/25	2/6/4	0.09
Survival (months)	34 ± 22	37 ± 22	21 ± 17	0.03
WBC (×10^9^ L)	8.10 ± 2.39	8.11 ± 2.31	8.06 ± 2.79	0.95
Lymphocytes (×10^9^ L)	2.22 ± 0.91	2.42 ± 0.88	1.53 ± 0.65	0.002
Monocytes (×10^9^ L)	0.50 (0.40–0.60)	0.50 (0.40–0.60)	0.49 (0.35–0.60)	0.28
Neutrophils (×10^9^ L)	4.91 ± 2.07	4.72 ± 1.95	5.60 ± 2.40	0.20
IMA (mABSU)	481 ± 49	472 ± 42	508 ± 64	0.035

6MWT: six-minute walk test; BMI: body mass index; DLCO: capacity for carbon monoxide; F: female; FEV_1_: forced expiratory volume in the 1st second; FVC: forced vital capacity; GERD: gastroesophageal reflux disease; M: male; TLC: total lung capacity; WBC: white blood cell.

**Table 2:** Univariate Cox regression showing the hazard ratios for the studied variables.

	HR	95% CI	*p*-Value
Age	0.9643	0.8789 to 1.0580	0.44
Gender	0.4393	0.0566 to 3.4119	0.43
BMI	1.0759	0.9375 to 1.2347	0.30
Smoking status	1.0847	0.3067 to 3.8364	0.90
Diabetes	2.6219	0.6871 to 10.005	0.16
Arterial hypertension	2.4741	0.7442 to 8.2246	0.14
Cerebrovascular diseases	0.8259	0.1062 to 6.4234	0.86
Atrial arrhythmias	2.3974	0.6467 to 8.8876	0.19
GERD	0.7503	0.2031 to 2.7713	0.67
FEV_1_ (%)	0.9765	0.9480 to 1.0058	0.11
FVC (%)	0.9720	0.9419 to 1.0031	0.08
FEV_1_/FVC	1.0671	0.9687 to 1.1755	0.19
TLC (%)	0.9587	0.9189 to 1.0001	0.051
DLCO (%)	0.9875	0.9564 to 1.0196	0.44
6MWT	0.9983	0.9947 to 1.0019	0.35
Stage	1.8409	0.8369 to 4.0494	0.13
Therapy	0.3993	0.1432 to 1.1135	0.08
WBC	0.9784	0.7375 to 1.2980	0.88
Lymphocytes	0.2671	0.1110 to 0.6427	0.003
Monocytes	0.0410	0.0007 to 2.2530	0.12
Neutrophils	1.1813	0.9219 to 1.5136	0.19
IMA	1.0154	1.0035 to 1.0275	0.01

6MWT: six-minute walk test; BMI: body mass index; DLCO: diffusion capacity for carbon monoxide; FEV_1_: forced expiratory volume in the 1st second; FVC: forced vital capacity; GERD: gastroesophageal reflux disease; IMA: ischemia-modified albumin; TLC: total lung capacity; WBC: white blood cell.

**Table 3:** Multivariate Cox regression model showing the hazard ratios for the studied variables.

	HR	95% CI	*p*-Value
FVC (%)	--	--	--
TLC (%)	0.9556	0.9116 to 1.0018	0.06
Therapy	--	--	--
Lymphocytes	0.3051	0.1349 to 0.6901	0.004
IMA	1.0118	1.0008 to 1.0230	0.036

FVC: forced vital capacity; TLC: total lung capacity; IMA: ischemia-modified albumin.

## Data Availability

The data presented in this study are available upon request from the corresponding author.

## References

[1] Raghu G., Collard H.R., Egan J.J., Martinez F.J., Behr J., Brown K.K., Colby T.V., Cordier J.F., Flaherty K.R., Lasky J.A. (2011). An official ATS/ERS/JRS/ALAT statement: Idiopathic pulmonary fibrosis: Evidence-based guidelines for diagnosis and management. Am. J. Respir. Crit. Care Med..

[2] King T.E., Pardo A., Selman M. (2011). Idiopathic pulmonary fibrosis. Lancet.

[3] Selvarajah B., Platé M., Chambers R.C. (2023). Pulmonary fibrosis: Emerging diagnostic and therapeutic strategies. Mol. Asp. Med..

[4] Fois A.G., Paliogiannis P., Sotgia S., Mangoni A.A., Zinellu E., Pirina P., Carru C., Zinellu A. (2018). Evaluation of oxidative stress biomarkers in idiopathic pulmonary fibrosis and therapeutic applications: A systematic review. Respir. Res..

[5] Paliogiannis P., Fois A.G., Collu C., Bandinu A., Zinellu E., Carru C., Pirina P., Mangoni A.A., Zinellu A. (2018). Oxidative stress-linked biomarkers in idiopathic pulmonary fibrosis: A systematic review and meta-analysis. Biomark. Med..

[6] Cheresh P., Kim S.J., Tulasiram S., Kamp D.W. (2013). Oxidative stress and pulmonary fibrosis. Biochim. Biophys. Acta.

[7] Winterbourn C.C. (2008). Reconciling the chemistry and biology of reactive oxygen species. Nat. Chem. Biol..

[8] Kamp D.W., Graceffa P., Pryor W.A., Weitzman S.A. (1992). The role of free radicals in asbestos-induced diseases. Free. Radic. Biol. Med..

[9] Balık Z.B., Balık A.R., Oğuz E.F., Erel Ö., Tunca M. (2023). Evaluation of Thiol Disulfide Homeostasis and Ischemia-Modified Albumin Levels as an Indicator of Oxidative Stress in Acne Vulgaris. Dermatol. Pract. Concept..

[10] Turan Ç., Şenormancı G., Neşelioğlu S., Budak Y., Erel Ö., Şenormancı Ö. (2023). Oxidative Stress and Inflammatory Biomarkers in People with Methamphetamine Use Disorder. Clin. Psychopharmacol. Neurosci..

[11] Bar-Or D., Lau E., Winkler J.V. (2000). A novel assay for cobalt-albumin binding and its potential as a marker for myocardial ischemia-a preliminary report. J. Emerg. Med..

[12] Bar-Or D., Winkler J.V., Vanbenthuysen K., Harris L., Lau E., Hetzel F.W. (2001). Reduced albumin-cobalt binding with transient myocardial ischemia after elective percutaneous transluminal coronary angioplasty: A preliminary comparison to creatine kinase-MB, myoglobin, and troponin I. Am. Heart J..

[13] Bakula M., Milicevic G., Bakula M., Kozic I., Rumenjak V., Dominkovic A. (2016). Kinetics of Ischemia-Modified Albumin Following Exercise-Induced Myocardial Ischemia. Clin. Lab..

[14] Sinha M.K., Vazquez J.M., Calvino R., Gaze D.C., Collinson P.O., Kaski J.C. (2006). Effects of balloon occlusion during percutaneous coronary intervention on circulating Ischemia Modified Albumin and transmyocardial lactate extraction. Heart.

[15] Bhagavan N.V., Lai E.M., Rios P.A., Yang J., Ortega-Lopez A.M., Shinoda H., Honda S.A., Rios C.N., Sugiyama C.E., Ha C.E. (2003). Evaluation of human serum albumin cobalt binding assay for the assessment of myocardial ischemia and myocardial infarction. Clin. Chem..

[16] Kaefer M., Piva S.J., De Carvalho J.A., Da Silva D.B., Becker A.M., Coelho A.C., Duarte M.M., Moresco R.N. (2010). Association between ischemia modified albumin, inflammation and hyperglycemia in type 2 diabetes mellitus. Clin. Biochem..

[17] Dayanand C., Vegi P.K., Lakshmaiah V., Kutty A. (2013). Association of ischemia modified albumin in terms of hypoxic risk with carbonylated protein, glycosylated hemoglobin and plasma insulin in type 2 diabetes mellitus. Int. J. Biotech. Biochem..

[18] Chawla R., Loomba R., Guru D., Loomba V. (2016). Ischemia Modified Albumin (IMA)—A Marker of Glycaemic Control and Vascular Complications in Type 2 Diabetes Mellitus. J. Clin. Diagn. Res. JCDR.

[19] Szulimowska J., Zalewska A., Taranta-Janusz K., Trocka D., Żendzian-Piotrowska M., Tomasiuk R., Maciejczyk M. (2023). Association of Ischemia-Modified Albumin (IMA) in Saliva, Serum, and Urine with Diagnosis of Chronic Kidney Disease (CKD) in Children: A Case-Control Study. Med. Sci. Monit. Int. Med. J. Exp. Clin. Res..

[20] Le Q.F., Liu J., Chen L. (2023). The value of serum lipoprotein-associated phospholipase A2, ischemia-modified albumin, and cystatin C in predicting coronary heart disease risk: A single center retrospective cohort study. Eur. Rev. Med. Pharmacol. Sci..

[21] Singh A., Pogorelić Z., Agrawal A., Muñoz C.M.L., Kainth D., Verma A., Jindal B., Agarwala S., Anand S. (2023). Utility of Ischemia-Modified Albumin as a Biomarker for Acute Appendicitis: A Systematic Review and Meta-Analysis. J. Clin. Med..

[22] Zhong C., Chen T., Shen Y., Zhang Y., Liu Y., Ning L. (2021). The effects of serum ischemia modified albumin on diagnosis of cerebral infarction and vertebral basilar artery stenosis. Brain Inj..

[23] Afrose D., Chen H., Ranashinghe A., Liu C.C., Henessy A., Hansbro P.M., McClements L. (2022). The diagnostic potential of oxidative stress biomarkers for preeclampsia: Systematic review and meta-analysis. Biol. Sex Differ..

[24] Brusasco V., Crapo R., Viegi G., American Thoracic Society, European Respiratory Society (2005). Coming together: The ATS/ERS consensus on clinical pulmonary function testing. Eur. Respir. J..

[25] Zinellu A., Paliogiannis P., Sotgiu E., Mellino S., Mangoni A.A., Zinellu E., Negri S., Collu C., Pintus G., Serra A. (2020). Blood Cell Count Derived Inflammation Indexes in Patients with Idiopathic Pulmonary Fibrosis. Lung.

[26] Achaiah A., Rathnapala A., Pereira A., Bothwell H., Dwivedi K., Barker R., Iotchkova V., Benamore R., Hoyles R.K., Ho L.P. (2022). Neutrophil lymphocyte ratio as an indicator for disease progression in Idiopathic Pulmonary Fibrosis. BMJ Open Respir. Res..

[27] Chen Y., Cai J., Zhang M., Yan X. (2022). Prognostic Role of NLR, PLR and MHR in Patients With Idiopathic Pulmonary Fibrosis. Front. Immunol..

[28] Bernardinello N., Grisostomi G., Cocconcelli E., Castelli G., Petrarulo S., Biondini D., Saetta M., Spagnolo P., Balestro E. (2022). The clinical relevance of lymphocyte to monocyte ratio in patients with Idiopathic Pulmonary Fibrosis (IPF). Respir. Med..

[29] Zinellu A., Collu C., Nasser M., Paliogiannis P., Mellino S., Zinellu E., Traclet J., Ahmad K., Mangoni A.A., Carru C. (2021). The Aggregate Index of Systemic Inflammation (AISI): A Novel Prognostic Biomarker in Idiopathic Pulmonary Fibrosis. J. Clin. Med..

[30] Marzec J.M., Nadadur S.S. (2022). Inflammation resolution in environmental pulmonary health and morbidity. Toxicol. Appl. Pharmacol..

[31] Rogers L.K., Cismowski M.J. (2018). Oxidative Stress in the Lung—The Essential Paradox. Curr. Opin. Toxicol..

[32] van der Vliet A., Janssen-Heininger Y.M.W., Anathy V. (2018). Oxidative stress in chronic lung disease: From mitochondrial dysfunction to dysregulated redox signaling. Mol. Asp. Med..

[33] Sarma J.V., Ward P.A. (2011). Oxidants and redox signaling in acute lung injury. Compr. Physiol..

